# An Integrative Network Science and Artificial Intelligence Drug Repurposing Approach for Muscle Atrophy in Spaceflight Microgravity

**DOI:** 10.3389/fcell.2021.732370

**Published:** 2021-09-16

**Authors:** Vidya Manian, Jairo Orozco-Sandoval, Victor Diaz-Martinez

**Affiliations:** Laboratory for Applied Remote Sensing, Imaging, and Photonics, Department of Electrical and Computer Engineering, University of Puerto Rico, Mayaguez, PR, United States

**Keywords:** muscle atrophy, network measures, random walk, graph neural network, random forest, gradient boosting, preferential attachment, link prediction

## Abstract

Muscle atrophy is a side effect of several terrestrial diseases which also affects astronauts severely in space missions due to the reduced gravity in spaceflight. An integrative graph-theoretic network-based drug repurposing methodology quantifying the interplay of key gene regulations and protein–protein interactions in muscle atrophy conditions is presented. Transcriptomic datasets from mice in spaceflight from GeneLab have been extensively mined to extract the key genes that cause muscle atrophy in organ muscle tissues such as the thymus, liver, and spleen. Top muscle atrophy gene regulators are selected by Bayesian Markov blanket method and gene–disease knowledge graph is constructed using the scalable precision medicine knowledge engine. A deep graph neural network is trained for predicting links in the network. The top ranked diseases are identified and drugs are selected for repurposing using drug bank resource. A disease drug knowledge graph is constructed and the graph neural network is trained for predicting new drugs. The results are compared with machine learning methods such as random forest, and gradient boosting classifiers. Network measure based methods shows that preferential attachment has good performance for link prediction in both the gene–disease and disease–drug graphs. The receiver operating characteristic curves, and prediction accuracies for each method show that the random walk similarity measure and deep graph neural network outperforms the other methods. Several key target genes identified by the graph neural network are associated with diseases such as cancer, diabetes, and neural disorders. The novel link prediction approach applied to the disease drug knowledge graph identifies the Monoclonal Antibodies drug therapy as suitable candidate for drug repurposing for spaceflight induced microgravity. There are a total of 21 drugs identified as possible candidates for treating muscle atrophy. Graph neural network is a promising deep learning architecture for link prediction from gene–disease, and disease–drug networks.

## Introduction

Drug discovery is an expensive process costing an average of $1.8 million per drug. Most drug discovery done on Earth is under a constant environment with a gravity value of 9.81 m/s^2^. Spaceflight in satellites and the International Space Station (ISS) provides a gravitational acceleration of 1 × 10^–6^ m/s^2^. This is referred to as microgravity which has direct and indirect effects on an organism. The direct effects are changes in weight, distortion and deformation of organelles, and other measurable changes. The indirect changes are those that occur prior due to microgravity. Bacterial virulence and increased genetic recombination have been observed in space thereby requiring increased concentrations of antibiotics for treatment. Spaceflight environment is conducive for drug discovery, as observed in an experiment conducted on spaceflight tested a molecule Amgn-0007 and sActRIIB for increasing bone mineral density in mice (Zea, 2015).

In addition to aging, muscle atrophy is slightly implicated in the etiology of chronic diseases such as diabetes, cancer, obesity, and muscular dystrophy (Kalyani et al., 2014; Muscular Dystrophy, n.d.).^1^ Muscle wasting also develops as a consequence of acquired immune deficiency syndrome (AIDS) (Dudgeon et al., 2006), neuromuscular disorders, and organ failure (cachexia) (Wyart et al., 2020; Rausch et al., 2021). Muscle wasting is the hallmark of cancer cachexia and is associated with serious clinical consequences such as physical impairment, poor quality of life, reduced tolerance to treatments, and shorter survival (Burckart et al., 2010). Muscle atrophy is a severe disabling clinical condition that is accompanied by cancer development in the pancreatic, lung, liver, and bladder (Bei and Xiao, 2017; Yang et al., 2018). Prolonged stay in spaceflight of up to 4 months can lead to a 17% loss of muscle mass. Muscle atrophy condition is accelerated in space due to microgravity by unloading of the muscles. Gene expression datasets have been analyzed using traditional fold change analysis and clustering methods for the identification of differentially regulated genes involved in muscle atrophy in mice flown in spaceflight (Horie et al., 2019a,b). Spaceflight simulation studies have shown differential expression of small number of microRNAs in the context of muscle physiology in response to loading (Rullman et al., 2016). Recent miRNA studies have shown that muscle degeneration with accelerated aging enhanced by exposure to space radiation and microgravity are driven by circulating miRNA and are being suggested as a potential biomarker (Malkani et al., 2020). But, advanced network analysis to identify causally related key target genes and their association with other diseases, and the application of Artificial Intelligence (AI) methods for identification of drugs suitable for treatment of muscle atrophy in spaceflight have not been performed.

Several treatments have been proposed and used for countering muscle atrophy in humans. Inhibition of a protein called myostatin has shown to result in an increase in muscle mass (Smith et al., 2020). The drug formeterol has been used for counteracting muscle atrophy in mice in spaceflight (Ballerini et al., 2020). There are many drug candidates that can be used for treating muscle atrophy, and the use of traditional methods for drug repurposing are time consuming due to the large volume of compounds that need to be tested. AI based methods have gained importance in this pandemic era for rapid, low-cost, and effective drug repurposing (Gysi et al., 2020). AI methods rely on the fact that drugs that target one disease can target another disease with similarly functioning protein–protein interaction networks. AI related methods are Machine Learning methods and/or Deep Learning (DL), a sub-branch of ML (Chen et al., 2020). ML methods such as Support Vector Machines (SVM), Random Forest (RF), and Gradient Boosting (Gboost) method have been used for drug repositioning to treat schizophrenia and anxiety disorders (Zhao and So, 2018). Employing ML based drug repositioning is a cost-effective way of automatizing the drug discovery process, and gaining deeper knowledge in the genetic causality of diseases, their associations, and planning preclinical trials for the selected drugs (Koromina et al., 2019; Réda et al., 2020). DL neural network architectures can explore a large amount of data, and search for similarities in several thousands of protein-protein interactions. If the input data is in the form of sequences, then Recurrent Neural Networks (RNN) are trained with the time-stamped data and used for prediction of drugs (Wang et al., 2020). Hybrid models that combine the power of Convolutional Neural Networks (CNN) and RNN have been used for drug repurposing (Xuan et al., 2019; Jarada et al., 2020). Gene protein and protein–protein interactions are generally depicted in the form of a graph, which have led to identifying disease networks and network medicine approaches for drug repurposing (Gysi et al., 2020). Network measures and evaluation metrics such as Area Under Receiver Operating Characteristic (AUROC) curves, and Area Under Precision and Recall (AUPR) have been used for network link prediction in drug discovery (Chen et al., 2018; Abbas et al., 2021). Network medicine uses graph representation for learning the patterns of protein–protein interactions. The SPOKE database (Nelson et al., 2021) is a heterogeneous knowledge graph connecting biological and clinical data from over 30 databases, that is used in this work in combination with transcriptomic datasets to create the inputs to the AI model. The Bayesian Markov blanket method applied to spaceflight transcriptomic datasets for muscle atrophy gives information on which genes are highly activated due to muscle unloading in spaceflight.

In this paper, we analyze spaceflight gene expression datasets for muscle atrophy using advanced network analysis methods and combine it with the power of AI for identifying drugs that can be repurposed for successful treatment of muscle atrophy. The rest of the paper is organized as follows. Section “Materials and Methods” presents the GeneLab datasets, and the methods used for drug repurposing, Section “Results” presents the knowledge graphs, and the link prediction results, section “Discussion” presents a discussion of the gene-disease associations, and disease-drug link predictions, and the “Conclusions” section presents the conclusions.

## Materials and Methods

This section describes the GLDS datasets used for mining, the SPOKE database, the network analysis methods, and the ML and AI methods for link prediction. Gene expression data were downloaded from NASA GeneLab repository. The datasets were preprocessed by NASA GeneLab.

### GLDS-4

Thymus lobes were extracted from young adult C57BL/6NTac mice at 8 weeks of age after exposure to spaceflight aboard the space shuttle STS-118 for a period of 13 days. Gene expression analysis demonstrate that spaceflight induces significant changes in the thymic mRNA expression of genes that regulate stress, glucocorticoid receptor metabolism, and T cell signaling activity (Lebsack et al., 2010). Key master regulators such as TGF-β1 coordinating systemic response of mice to spaceflight microgravity and/or space radiation were identified in Beheshti et al. (2018).

### GLDS-244

A cohort of healthy mice was implanted with subcutaneous nanofluidic delivery system (nF) of formoterol (FMT), a β2-adrenergic receptor agonist for therapeutic treatment of skeletal muscle loss. The mice were subjected to spaceflight microgravity on ISS for 29 and 56 days before euthanizing. RNA sequencing analysis of thymus tissues showed that nF-FMT treatment mass loss in comparison to control mice (Ballerini et al., 2020).

### GLDS-245

Liver tissue was extracted from the same cohort of mice used in GLDS-244 experiment. RNA sequence data was obtained from liver preserved in liquid nitrogen after dissection and stored at –80°C. RNA sequencing analysis of thymus tissues was done.

### GLDS-246

A cohort of forty 32-weeks-old female C57BL/6NTac mice were either sham operated or implanted with vehicle or treatment-filled nDS, launched in two Transporters (20 mice per Transporter) on SpaceX-13. They were transferred to Rodent Habitats onboard the ISS, and maintained in microgravity. After 56 days, they were euthanized on the ISS and RNA samples from spleen tissue was extracted and sequencing analysis was performed.

### GLDS-288

The spleens and lymph nodes were analyzed from mice flown aboard the ISS in orbit for 35 days, as part of a Japan Aerospace Exploration Agency mission. The mice were exposed to 1 g microgravity in the ISS. Paired end sequencing (PE36bp) was performed with NextSeq500. Whole-transcript cDNA sequencing (RNASeq) analysis of the spleen suggested that erythrocyte-related genes regulated by the transcription factor GATA1 and Tal1 were significantly down-regulated in ISS (Horie et al., 2019b).

### GLDS-289

Twelve C57BL/6 J male mice (8-week-old for MHU-1 and 9-week-old for MHU-2) in transportation cage units (TCU) were launched aboard the SpaceX rocket from the KSC and transported to the ISS. After one month in spaceflight, RNA sequencing analysis showed a significantly reduced expression of cell cycle-regulating genes, resulting in reduced size of thymus. However, exposure to 1 × g alleviated the impairment of thymus homeostasis induced by spaceflight (Horie et al., 2019a).

### Gene Regulatory Network Inferencing Using Incremental Association Markov Blanket Method

In genomics, genome to phenome analysis, and transcriptional regulatory analysis are facilitated by construction of Gene Regulatory Networks (GRNs) from gene expression datasets. The GRNs also show causal relations between the genes. Traditionally, causal relations are difficult to infer and require careful application of experimental interventions. However, causal relations can be discovered by statistical analysis of purely observational data, which is known as causal structure learning (Anand, 2009). Using Markov property, a gene is conditionally independent of all other genes except its parents, children, and children’s parent variables (genes). Causal relationships are useful for combining omics data with Genome Wide Association Studies (GWAS), for inferring relationships between genotype and phenotype (Ainsworth et al., 2017).

The method used for causal relation inferencing used here is the Markov Blankets (MB) method and Bayesian Network (BN) learning (Tsamardinos et al., 2003; Ram and Chetty, 2011; Syed Sazzad et al., 2020). Joint conditional probabilities are represented by a graph in a Bayesian network, the nodes (genes) are connected by Markov property which states that a node is conditionally independent of its non-descendants, given its parents. Applying the faithfulness condition, the IAMB of any node (gene) in a BN is the set of parents, children, and spouses (the other parents of their common children) of the gene. In our case, each gene is a variable with a series of expression values. The Markov blanket of a gene X is the smallest set MB(X) containing all genes carrying information about X that cannot be obtained from any other gene. Association measures and conditional independent tests are applied to identify the strongly relevant genes (Pellet and Elisseeff, 2008; Bui and Jun, 2012). Hence, MB(T) is a causal structure learning algorithm useful for the discovery of regulatory interactions among genes from gene expression data. Here, MB is used to construct GRNs for regulatory relationship between genes/proteins.

### Gene Disease Knowledge Graph Using SPOKE

Gene disease associations are important as the key genes of muscle atrophy are also affected by other diseases which can turn out to be lethal when transferred to the next generation. Hence, it is vital to predict which new disease can occur because of the higher activity of particular genes in the GRNs for muscle atrophy. In order to obtain the gene disease associations, we use the Scalable Precision Medicine Knowledge Engine (SPOKE), which is a large heterogeneous network containing multiple types of biological data capturing the essential structure of biomedicine and human health for discovery (Scalable Precision Medicine Knowledge Engine, n.d.). The maximally regulated genes identified from the GRNs are input to the SPOKE which generates all the diseases associated with these key genes obtained from the GRNs for muscle atrophy. These associations are used to construct the Gene Disease Knowledge Graph (GDKG).

### Network Measures

We define a network using a graph based representation. Formally, a graph is a pair of sets G: = (V,E) where | V| is the set of vertices (molecules, genes, proteins, nodes, points) and | E| is the set of edges, which is an ordered pair of V. The graph (V, E, o, t) is called directed, if directed edges are allowed, i.e., not all edges have reverse edges as members of E. In a directed graph, G = (V,E,o,t), the edges are e(u,v) ϵ E, the origin of e is denoted by o and the terminal v is denoted by t(v). In a network G = (V,E), for a node u, Γ(u) = {v| (u,v)ϵE} represents the set of neighbors of node u. The link prediction task in a network G = (V,E) is to determine whether there is or will be a link e(u,v) between a pair of nodes u and v, where *u*,*v* ∈ *V*, and *e*(*u*,*v*)∉*E*. Similarity measures computed from neighborhoods in a graph are widely used in link prediction algorithms (Abbas et al., 2021). Random walks have been used for link prediction. Random walk methods efficiently explore neighborhoods of a node to determine a path from a starting node to a terminal node. Probabilities are usually used to select the next neighboring node in the path. Biomolecular networks are complex and random walks are an efficient way for exploring them (Costa and Travieso, 2007; Janwa et al., 2019). A semi-supervised scalable feature learning method is proposed in Grover and Leskovec (2016), where the authors develop a family of biased random walks resulting in a flexible search space of nodes for link prediction. We have used this method to obtain the highest ranked nodes for possible links between muscle atrophy genes and their associated diseases. Apart from random walk, we have computed the preferential attachment network measure to obtain possible gene–disease and disease–drug associations. Preferential Attachment is the multiplication of the degrees of nodes u and v: *PA*(*u*,*v*) = |Γ(*u*)||Γ(*v*)|

### Graph Neural Network for Prediction of Gene-Disease Associations

A deep Graph Neural Network (GNN) architecture consisting of multiple layers and hundreds of nodes is constructed and takes as input the GDKG constructed as described in section “Gene Disease Knowledge Graph Using SPOKE.” This graph G = (V,E) is multimodal and heterogeneous with N nodes vi ϵ V is the set of nodes representing proteins or genes, and diseases. The edges E represents gene-disease associations. The link prediction task is to predict whether there will, is, or will be a link e(u,v) between a pair of nodes u and v, where u, v ϵV and *e*(*u*,*v*)∉*E*. A link prediction problem is setup on the GDKG representation for identifying links between genes and the diseases associated with it. The GNN is a three layer model. The edge features of the GDKG are the input to the input layer of the GNN. The hidden layer consists of 300 neurons with “tanh” activation function. Limited-memory Broyden–Fletcher–Goldfarb–Shanno (lbfgs) solver from the sktlearn library is used for link prediction. It approximates the second derivative matrix updates with gradient evaluations. It stores only the last few updates, so it saves memory. The output of the GNN is a matrix consisting of new predicted edges.

### Random Forest Method

The RF is a classifier using the ensemble learning algorithm on a multitude of decisions trees constructed at training time. It trains decision trees using random sampling with replacement. For each node in the base decision tree, random forest randomly chooses an attribute subset including k (k ≤ m) attributes from the attribute set of the node (including m attributes), and then, chooses the best attribute from the subset to split samples (the optimal judgment is usually based on the minimum of a Gini index). The split process will be repeated until the split termination condition is satisfied (generally, the Gini index is small enough), and the model integrated by multiple decision trees is a random forest (Wu et al., 2018). Each tree emits a prediction, and the class with the most votes becomes the model’s prediction. It is based on the principle that many uncorrelated models (trees) operating as a committee will outperform any of the individual constituent models.

### Gradient Boosting Classifier

The Gboost classifier is also an ensemble learning method similar to random forest except that it trains one tree at a time. This additive model (ensemble) works in a forward stage-wise manner, introducing a weak learner to improve the shortcomings of existing weak learners (Li et al., 2016). In Gboost, shortcomings are identified by gradients. Whereas in Adaboost, shortcomings are identified by high-weight data points. Both high-weight data points and gradients tell us how to improve our model. RF combine results at the end of the process (by averaging or “majority rules”) while Gboost combines results along the way. Gboost is not a best method if there is lot of noise in the data, as it results in overfitting. The parameters are harder to tune than RF.

### Disease Drug Link Prediction

The top ranked disease associations from the GDKG that have highest probability of predicted and existing links are selected. For each of these disease up to ten drugs are chosen from the DrugBank database (Drugbank online, n.d.).^2^ There are multiple drugs that are used for several diseases. An adjacency matrix with rows for diseases and columns for drugs is constructed. The Disease Drug Knowledge Graph (DDKG) is generated and the link prediction algorithm is run on this graph. This results in top ranked drugs with highest probability that can be repurposed for muscle atrophy. The choice of the best drug also depends on the diagnostics and prognostics of the disease, hence the most prevalent comorbidities with muscle atrophy is considered for the drug selection. Drug selection is also a very sensitive process and requires clinical intervention, hence, we provide a list of drugs that may be considered for treatment of this condition in spaceflight.

**FIGURE 1:**
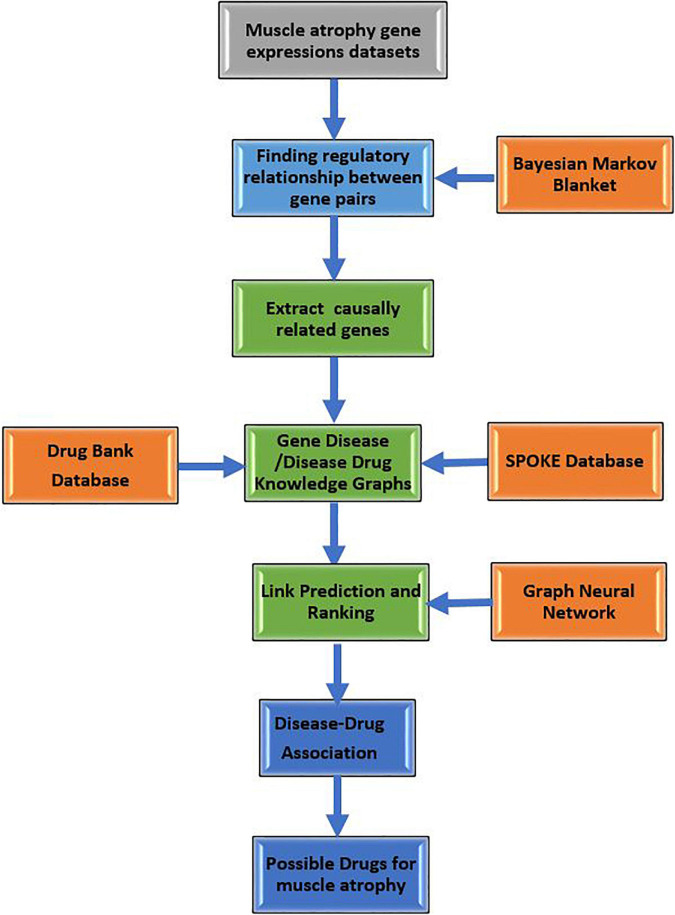
Flow diagram showing sequence of steps followed for constructing GDKG, DDKG, and link prediction for drug repurposing to treat muscle atrophy in spaceflight microgravity.

Figure 1 shows the sequence of steps followed for constructing GDKG, DDKG, and link prediction for drug repurposing in muscle atrophy. The predicted drugs have the highest probability for muscle atrophy treatment in spaceflight. Once the DDKG is constructed any machine learning approach for link prediction can be used for predicting probable drugs. The network feature extraction method used here is based on random walks, which can be replaced with other local or global graph similarity based indices such as common neighbors, Jaccard index, Sorensen index, preferential attachment, Adamic-Adar index, resource allocation index, hub promoted index, Leicht-Holme-Newman index, parameter dependent index, local affinity structure index, individual attraction index, mutual information index, functional similarity weight, and local neighbors link index, Katz index, and page rank (Fire et al., 2011; Mutlu et al., 2020). In this case, we found random walk and preferential attachment to give better results than the other features.

### Metrics for Evaluation of Link Prediction Methods

There are several measures to evaluate the performance of link prediction methods. The Receiver Operating Curve (ROC) represents the performance trade-off between true positive and false positives at different decision boundary thresholds. AUROC is the Area Under the Receiver Operating Characteristics (AUROC) value, which is the area under the plot between True Positive Rate (TPR) and the False Positive Rate (FPR). It represents the trade-of between TP and FP prediction rates. The TPR is also known as sensitivity, recall, or probability of detection. AUROC measures the separability of the classifier and is therefore a vital metric (Yang et al., 2015).

### Computational Network Measures

The network measures used to analyze the GDKG and DDKG networks include spectral gap, girth or diameter, and density. Measures computed on the gene nodes and drug nodes are degree distribution, neighborhood connectivity, and subgraph centrality (Biggs, 1993).

Spectral gap: For a graph G, the Laplacian eigenvalues can be ordered as 1 = | λ_1_| ≥ | λ_2_| ≥ ⋅⋅⋅ ≥ | λn| (G may be directed or undirected, weighted or unweighted, simple or not). The Spectral gap is defined as: δ_λ_ = | λ_1_| – | λ_2_|. By normalizing the Laplacian matrix of G, the eigenvalues are λ_1_ ≥ λ_2_ ≥ ⋅⋅⋅ ≥ λn > 0, and the Laplacian spectral gap will be: δ_λ_ = 1 – | λ_2_|. The spectral gap is also known as a random walk, in terms of this concept λ_2_ is the most important eigenvalue. Note that if the spectral gap is 0, which means λ_2_ = 1 [Γ is not (strongly) connected or if Γ is bipartite], this means a typical random walk will not converge to a unique distribution or dominant eigenvector. As long as the spectral gap is greater than 0, which means | λ_2_| < 1, then the random walk converges to a unique dominant eigenvector, and the spectral gap measures the rate of convergence, the larger the spectral gap (the smaller| λ_2_|), the better the network flow [large h(G), diffusion, mixing, random walk, expansion, sparsity, and other highly desirable properties of the network G].

Girth of a graph is the smallest positive integer *r* such that Trace(A^r^) > 0. Let d = d(G) be the smallest integer (if it exists) so that for every pair of vertices (u,v) there is a walk of length at most d from u to v. Then d(G) is called the *diameter* or maximum eccentricity of the graph G.

Density of a graph is the ratio between the number of edges and the number of possible edges. Density is a measure of the compactness of a module (subnetwork) and measures the connectivity strength of pairs of genes in the module (Hussain Ahmed et al., 2020).

The clustering coefficient models the degree of clustering of a subset of nodes. A node is selected, and we see how connected the node is with other nodes that are also connected to it. The clustering coefficient is used to characterize network modularity, which is a strength of measure of a network division into modules or groups.

Degree distribution is the number of neighbors connected to a node; in other words, it is the number of edges incident on a node. The degree distribution can give information about the structure of a network. The networks can be directed or undirected. In the undirected case, the degree of node *i* is the number of connections it has, and it can be represented as an adjacency matrix, with the sum over all nodes. For directed graphs, there are two types of degree distributions: in-degree, which is the number of connections entering the node, and out-degree, which is the number of outgoing connections. In this case, the degree distribution is computed for the genes in the GDKG and for the drugs in the DDKG.

Subgraph centrality of a node is a weighted sum of closed walks of different lengths in the network starting and ending at a node. Centrality measures are used widely in biological networks to infer protein-protein interactions and identify essential proteins (Opsahl et al., 2010).

### Implementation

The GRN inferencing method using MB is implemented in R. This method is used to construct the GRN’s for each of the spaceflight muscle atrophy datasets. The GDKG construction is done using SPOKE database and its adjacency matrix is created in MS Excel. The drug disease adjacency matrix is first constructed in MS Excel after downloading the drugs for each disease from drug bank. Cytoscape is used to visualize the networks. Exhaustive search method from the GridSearchCV library is used to estimate the best parameters for the link prediction methods. For the gene disease link prediction, the parameters chosen for the RF method are: depth of 15 for the RF with 500 estimators, and a learning rate of 0.2 for Gboost method. The GNN is a deep neural network with 10 layers consisting of 100 hidden nodes in each layer, it uses “relu” for activation, and Adam solver. For disease drug link prediction, the estimated parameters are a depth of 5 for the RF method with 500 estimators, and a learning rate of 0.2 for the Gboost method. The GNN has 10 layers with 100 hidden nodes in each layer, uses “relu” for activation, and Lbfgs solver. The GridSearchCV library also estimates the best number of split for cross validation, as well. In our implementation, we have chosen 10-fold cross validation. The computation of network features, and graph features are implemented in Python using the libraries networkX, node2vec, pandas, numpy, and sklearn. The implementations are available in github.^3^

## Results

Results of GRN inferencing, knowledge graph construction, and the training and validation of link prediction methods are presented below.

### GRN Inferencing and Construction of Knowledge Graphs

**FIGURE 2:**
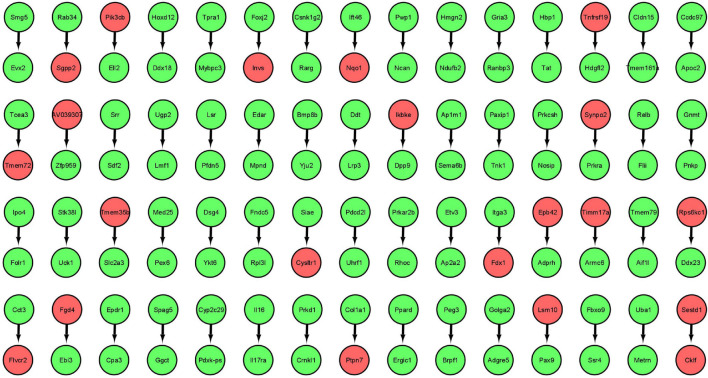
Markov Blanket Gene Regulatory Network for GLDS-246 dataset. Red colored circles are genes with higher regulatory activity selected for constructing the GDKG.

**FIGURE 3:**
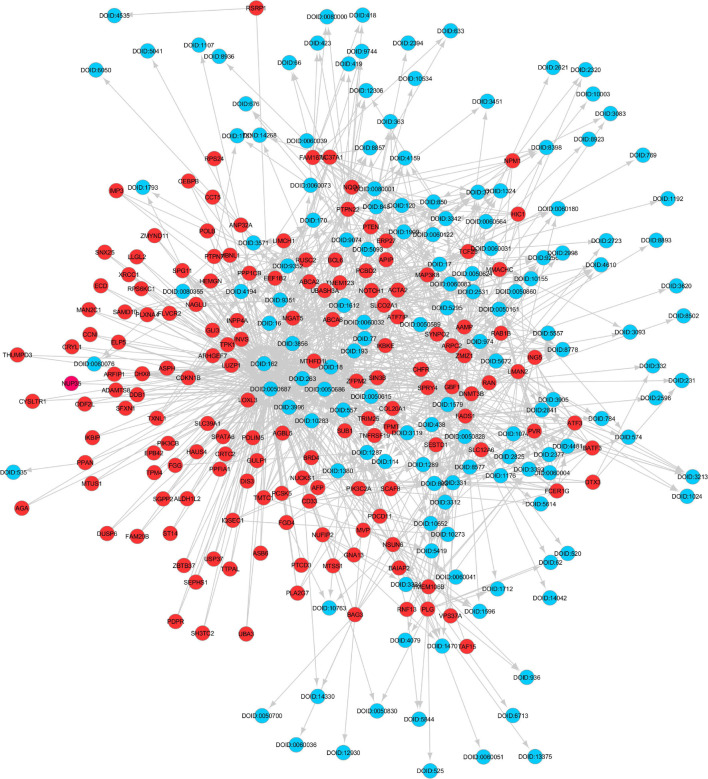
Gene Disease Knowledge Graph constructed from muscle atrophy genes and their associations with diseases identified from the SPOKE database. The red colored circles are the gene nodes and the blue colored circles are disease nodes.

**FIGURE 4:**
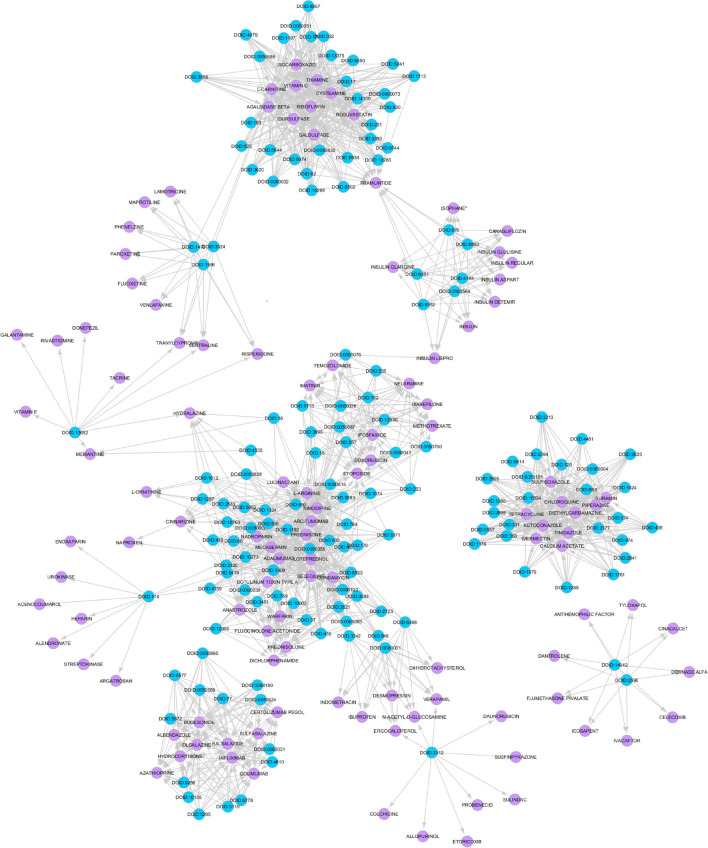
Disease Drug Knowledge Graph constructed from muscle atrophy related diseases and drugs used for their treatment obtained from the drug bank database. The blue colored circles are the disease nodes and the purple colored circles are the drugs.

The gene expression values corresponding to spaceflight experiments are extracted from the excel files for the six GeneLab datasets and input to the MB GRN inferencing method. The number of values range from three to eight. Figure 2 shows the MB GRN for GLDS-246 dataset. Table 1 gives the list of the common genes identified from the GRNs that are highly activated due to muscle atrophy in spaceflight microgravity from the GLDS-4, 244, 245, 246, 288, and 289 datasets. Red nodes are genes with higher regulatory activity for muscle atrophy selected for constructing the GDKG. Figure 3 shows the GDKG constructed from the highly activated genes from the six datasets and the SPOKE database. Figure 4 shows the complete DDKG. Table 2 lists the diseases identified from the GDKG. The GDKG in matrix notation is of dimension 299 × 1195, where 299 is the number of nodes and 1,195 is the number of edges.

**TABLE 1:** Maximally regulated genes for muscle atrophy from the spaceflight Genelab datasets GLDS-4, GLDS-244, GLDS-245, GLDS-246, GLDS-288, and GLDS-289 selected by Markov Blanket network analysis.

Gene name	Degree distribution	Neighborhood connectivity	Subgraph centrality
AAMP	16	19.6875	1,066,536
ABCA2	7	63.28571	2,032,188
ABCA6	12	40.08333	2,517,512
ACTA2	13	33	1,265,017
ADAMTS8	2	129	483,438.2
AFP	6	59.5	1,090,336
AGA	2	129	483,438.2
AGBL5	7	63.28571	2,032,188
ALDH1L2	2	129	483,438.2
ANP32A	8	44.375	1,095,830
APIP	6	15	51,842.77
ARFIP1	2	129	483,438.2
ARHGEF7	7	63.28571	2,032,188
ARPC2	16	19.6875	1,066,536
ASB6	2	129	483,438.2
ASPH	2	129	483,438.2
ATF3	17	10	204,496.1
ATF7IP	23	27.6	3,275,739
BAG3	11	31.36364	839,991.3
BAIAP2	5	55	543,055.9
BATF3	17	10	204,496.1
BCL6	8	56.125	2,101,026
BRD4	7	63.28571	2,032,188
CCNI	2	129	483,438.2
CCT5	2	129	483,438.2
CD33	6	52.66667	697,061.2
CDKN1B	13	33.84615	1,540,483
CEBPB	3	14.66667	8,307.823
CHFR	8	51.57143	1,029,718
COL20A1	2	129	483,438.2
CRTC2	8	59.5	1090336
CRYL1	2	129	483,438.2
CYSLTR1	2	129	483,438.2
DDB1	2	129	483,438.2
DHX8	7	63.28571	2,032,188
DIS3	7	63.28571	2,032,188
DNMT3B	16	40.61538	2,985,855
DTX3	5	11.4	18,321.32
DUSP6	2	129	483,438.2
ECD	2	129	483,438.2
EEF1B2	8	47	1,208,616
ELP5	2	129	483,438.2
EPB42	2	129	483,438.2
ERP27	7	44.85714	686,034.2
FADS1	46	16.34091	4,995,617
FAM167A	13	6.615385	49,473.38
FAM20B	2	129	483,438.2
FCER1G	11	12.81818	141,251.6
FGD4	5	58	592,697.7
FGG	3	92.33333	551,896.8
FLVCR2	2	129	483,438.2
GBF1	22	27	3,570,302
GLI3	13	38.07692	2,695,724
GNA13	4	81.25	875,946
GULP1	2	129	483,438.2
HAUS4	2	129	483,438.2
HEMGN	7	65.6	906,075.6
HIC1	8	11.25	43,296.84
IKBIP	2	129	483,438.2
IKBKE	11	13	134,489.2
IMP3	3	14.66667	8,307.823
ING5	11	12.72727	194,840.4
INPP4A	2	129	483,438.2
INVS	7	63.28571	2,032,188
IQSEC1	5	58.4	625,550.6
LIMCH1	12	41.08333	2,669,384
LLGL2	2	129	483,438.2
LMAN2	24	22.45833	2,625,946
LOXL3	2	129	483,438.2
LUZP1	7	63.28571	2,032,188
MAP3K8	6	52.5	811,120
MBNL1	11	44.55556	1,288,207
MGAT5	12	34.45455	1,180,487
MMACHC	7	44	701,632.3
MTHFD1L	13	40.76923	2,703,941
MTSS1	5	57.8	587,272.6
MTUS1	2	129	483,438.2
MVP	5	58.2	651,412.9
NAGLU	5	60.4	617,943.7
NOTCH1	34	21.58065	5,186,101
NPM1	10	10.6	76,055.24
NQO1	9	11.22222	50,685.27
NSUN6	15	24.4	920,237.7
NUCKS1	13	34.30769	1,624,638
NUFIP2	5	55	543,055.9
NUP35	2	129	483,438.2
ODF2L	2	129	483,438.2
PCBD2	15	21.4	1,100,177
PCSK5	3	87.66667	510,939.4
PDCD11	10	47.7	2,313,937
PDLIM5	8	59.5	1,090,336
PDPR	2	129	483,438.2
PIK3C2A	9	52	2,218,154
PIK3CB	2	129	483,438.2
PLA2G7	4	71.75	599,936.5
PLG	18	7.5	97,555.15
PLXNA4	2	129	483,438.2
POLB	2	129	483,438.2
PPAN	3	86.33333	485,964.5
PPFIA1	7	63.28571	2,032,188
PPP1CB	7	56.16667	954,019.9
PTCD3	2	129	483,438.2
PTEN	36	18.77778	5,440,125
PTPN22	36	16.76471	2,848,757
PTPN7	5	42.2	404,444.5
PVR	12	11.91667	142,451.7
RAB1B	6	10.66667	25,252.28
RAN	13	28.30769	1,013,655
RNF13	4	9	5,753.418
RPS24	2	129	483,438.2
RPS6KC1	2	129	483,438.2
RSRP1	2	6	1,857.078
RUSC2	7	63.28571	2,032,188
SAMD10	3	14.66667	8,307.823
SCAF8	11	45.72727	2,543,016
SEPHS1	2	129	483,438.2
SESTD1	9	50.55556	2,117,183
SFXN1	2	129	483,438.2
SGPP2	2	129	483,438.2
SH3TC2	2	129	483,438.2
SIN3B	7	63.28571	2,032,188
SLC12A6	8	56.625	2,121,072
SLC37A1	18	23.83333	1,425,904
SLC39A1	6	59.5	1,090,336
SLCO2A1	13	33.41667	1,510,348
SNX25	2	129	483,438.2
SPATA6	2	129	483,438.2
SPG11	7	63.28571	2,032,188
SPRY4	22	24.42857	2,141,129
ST14	2	129	483,438.2
SUB1	7	34.42857	498,913.4
SYNPO2	12	40.08333	2,517,512
TAF15	1	10	953.6951
TCF25	24	19.5	1,637,001
THUMPD3	2	129	483,438.2
TMEM106B	7	8	16,732.19
TMEM123	6	59.5	1,090,336
TMTC1	8	56.625	2,121,072
TNFRSF19	12	38.7	1,291,127
TPK1	6	67	945,841.4
TPM4	2	129	483,438.2
TPMT	10	28.8	663,140.7
TRIM25	4	14.25	42,289.27
TTPAL	2	129	483,438.2
TXNL1	5	60.4	617,943.7
UBA3	2	129	483,438.2
UBASH3A	23	23.7619	2,102,919
USP37	2	129	483,438.2
VPS37A	4	12.75	16,391.32
XRCC1	2	129	483,438.2
ZBTB37	2	129	483,438.2
ZFPM2	17	32.47059	3,141,241
ZMIZ1	40	17.97368	4,230,617
ZMYND11	2	129	483,438.2

*The network measurements computed for these genes from the GDKG is given in columns 2–4.*

**TABLE 2:** Key diseases in which the genes that are maximally regulated in muscle atrophy are involved.

Disease Names				
Gout	Asthma	Cancer	Adenoma	Colitis
Leprosy	Obesity	Alopecia	Glaucoma	Leukemia
Lymphoma	Melanoma	Myopathy	Myositis	Rhinitis
Syndrome	Vitiligo	Arthritis	Carcinoma	Pemphigus
Psoriasis	Tauopathy	Dermatitis	Narcolepsy	Vasculitis
Cholangitis	Eye disease	Lung cancer	Scleroderma	Skin cancer
Bone disease	Hair disease	Hypertension	Liver cancer	Lung disease
Nose disease	Skin disease	Brain disease	Breast cancer	Dental caries
Endometriosis	Heart disease	Hypotrichosis	Kidney cancer	Larynx cancer
Liver disease	Lymphadenitis	Mood disorder	Mouth disease	Neuroblastoma
Overnutrition	Prion disease	Schizophrenia	Skin melanoma	Tooth disease
Acute leukemia	Aortic disease	Artery disease	Breast disease	Cardiomyopathy
Celiac disease	Hypothyroidism	Kidney disease	Kidney failure	Lung carcinoma
Osteoarthritis	Ovarian cancer	Skin carcinoma	Sleep disorder	Spinal disease
Stomach cancer	Uterine cancer	B-cell lymphoma	Benign neoplasm	Crohn’s disease
Genetic disease	Gonadal disease	Graves’ disease	Nephrolithiasis	Ovarian disease
Prostate cancer	Stomach disease	Synucleinopathy	Thoracic cancer	Uterine disease
Allergic disease	Bipolar disorder	Breast carcinoma	Cell type cancer	Kawasaki disease
Muscular disease	Pancreas disease	Prostate disease	Thoracic disease	Vascular disease
Allergic rhinitis	Bile duct disease	Bronchial disease	Colorectal cancer	Diabetes mellitus
Esophageal cancer	Inner ear disease	Intestinal cancer	Laryngeal disease	Leukocyte disease
Lymphoid leukemia	Mental depression	Monogenic disease	Nutrition disease	Pancreatic cancer
Rheumatic disease	Testicular cancer	Autoimmune disease	Bipolar I disorder	Cognitive disorder
Colorectal adenoma	Endometrial cancer	Esophageal disease	Hematologic cancer	Intestinal disease
Lymph node disease	Multiple sclerosis	Prostate carcinoma	Psychotic disorder	Sjogren’s syndrome
Temporal arteritis	Testicular disease	Ulcerative colitis	Alzheimer’s disease	Androgenic alopecia
Atrial fibrillation	Gallbladder disease	Heart valve disease	Laryngeal carcinoma	Lupus erythematosus
Nicotine dependence	open-angle glaucoma	Organ system cancer	Ovarian dysfunction	Parkinson’s disease
Aortic valve disease	Basal cell carcinoma	Bullous skin disease	Esophageal carcinoma	Immune system cancer
Low tension glaucoma	Motor neuron disease	Nasal cavity disease	non-Hodgkin lymphoma	Pancreatic carcinoma
Rheumatoid arthritis	Substance dependence	Systemic scleroderma	Thyroid gland cancer	Aortic valve stenosis
Biliary tract disease	Demyelinating disease	Disease of metabolism	Endogenous depression	Hepatobiliary disease
Immune system disease	Muscle tissue disease	Myocardial infarction	Nervous system cancer	Thyroid gland disease
Urinary system cancer	angle-closure glaucoma	Ankylosing spondylitis	Autoimmune thyroiditis	Chronic kidney disease
Dilated cardiomyopathy	Endocrine gland cancer	Large intestine cancer	Nasopharyngeal disease	Nervous system disease
Sclerosing cholangitis	Sensory system disease	Urinary system disease	Auditory system disease	Cerebrovascular disease
Coronary artery disease	Lymphatic system cancer	Squamous cell carcinoma	Thyroid gland carcinoma	Autism spectrum disorder
Disease of mental health	Endocrine system disease	Heart conduction disease	Intrinsic cardiomyopathy	Lymphatic system disease
Obstructive lung disease	Photosensitivity disease	Type 1 diabetes mellitus	Type 2 diabetes mellitus	Viral infectious disease
Autosomal genetic disease	Bone inflammation disease	Cell type benign neoplasm	Connective tissue disease	Creutzfeldt-Jakob disease
Major depressive disorder	Neurodegenerative disease	Peripheral artery disease	Polycystic ovary syndrome	Reproductive organ cancer
Respiratory system cancer	Teeth hard tissue disease	Acquired metabolic disease	Autosomal dominant disease	Glucose metabolism disease
Inflammatory bowel disease	Intestinal benign neoplasm	Respiratory system disease	Sensorineural hearing loss	substance-related disorder
Disease by infectious agent	Hepatobiliary system cancer	Integumentary system cancer	Reproductive system disease	Testicular germ cell cancer
Acute lymphoblastic leukemia	Bacterial infectious disease	Chronic lymphocytic leukemia	Disease of anatomical entity	Hematopoietic system disease
Integumentary system disease	Organ system benign neoplasm	Plantar fascial fibromatosis	Systemic lupus erythematosus	Amyotrophic lateral sclerosis
Cardiovascular system disease	Central nervous system cancer	Juvenile rheumatoid arthritis	Marginal zone B-cell lymphoma	Central nervous system disease
Gastrointestinal system cancer	Male reproductive organ cancer	Musculoskeletal system disease	Primary angle-closure glaucoma	Carbohydrate metabolism disease
Gastrointestinal system disease	Lower respiratory tract disease	Specific developmental disorder	Upper respiratory tract disease	Female reproductive organ cancer
Male reproductive system disease	Pervasive developmental disorder	Primary immunodeficiency disease	Autonomic nervous system neoplasm	Central nervous system vasculitis
Disease of cellular proliferation	Laryngeal squamous cell carcinoma	Peripheral nervous system disease	Female reproductive system disease	Peripheral nervous system neoplasm
Abdominal obesity-metabolic syndrome	Primary bacterial infectious disease	Autoimmune disease of exocrine system	Chronic obstructive pulmonary disease	Abdominal obesity-metabolic syndrome 1
Autoimmune disease of endocrine system	Developmental disorder of mental health	Gastrointestinal system benign neoplasm	Attention deficit hyperactivity disorder	Autoimmune disease of the nervous system
estrogen-receptor negative breast cancer	Autoimmune disease of cardiovascular system	Autoimmune disease of central nervous system	Autoimmune disease of gastrointestinal tract	Autoimmune disease of musculoskeletal system
Human immunodeficiency virus infectious disease	Autoimmune disease of skin and connective tissue			

*Identified from the SPOKE GDKG gene–disease network.*

### Training and Validation of Link Prediction Methods

**FIGURE 5:**
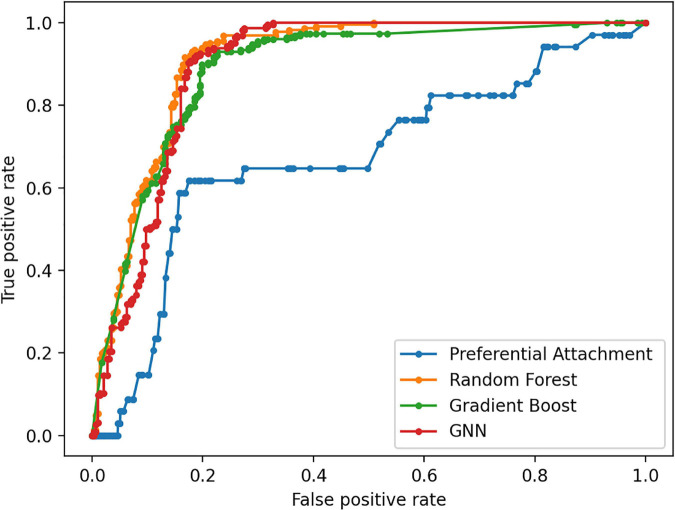
Receiver Operating Characteristic curves for link prediction between genes differentially regulated in muscle atrophy and diseases in the GDKG using PA, RF, Gboost, and GNN methods.

**FIGURE 6:**
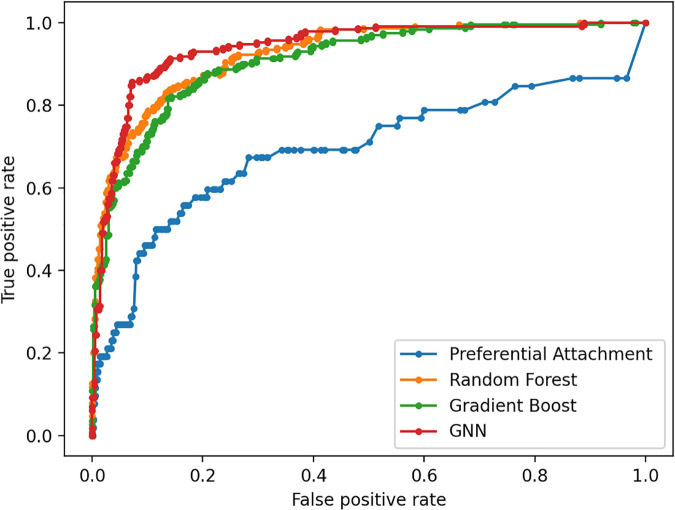
Receiver Operating Characteristic curves for link prediction between muscle atrophy related diseases and drugs used for their treatment in the DDKG using PA, RF, Gboost and GNN methods.

The Preferential Attachment (PA) method outputs the predicted links from the GDKG and DDKG matrices. These matrices are divided into training and validation sets. The training network is of size 299 × 298 which is input to the random walk network feature extraction method. The input matrix to the three link prediction methods of RF, Gboost, and GNN is a network measure matrix of dimension 2199 × 100, where 2,199 is the number of pairs of nodes, and 100 is the number of random walk features. Overall, the three link prediction methods perform better than PA method. The output of all the link prediction methods is a matrix of nodes and edges with a “1” indicating new edge between the node pairs. If an edge does not exist originally or after link prediction, that entry remains a “0.” Table 3 ranks the top muscle atrophy gene–disease associations based on a probability greater than 90% of link prediction using GNN. The most common disease associated with muscle atrophy are cancer, diabetes, and neural diseases. Table 4 lists the commonly used drugs for these diseases. There are about 180 drugs mentioned in the drug bank database as recommended treatment for the diseases mentioned in Table 2 which overlap with muscle atrophy condition. Table 5 lists 40 diseases with links to 21 drugs obtained from link prediction with probabilities higher than 80%. Some of these drugs treat more than one disease. Further fewer drugs can be selected by choosing a higher threshold for prediction probability. Table 6 lists the network measures computed for the 21 top ranked drugs in the DDKG. Table 7 lists the network measures for the GDKG and DDKG networks. Table 8 shows the True Positive, True Negative, False Positive, and False Negative for each of the link prediction methods for the GDKG and DDKG networks. Figures 5, 6 show the ROC curves for the GDKG and the DDKG link prediction, respectively. As can be seen the GNN has higher AUROC, followed by the RF method. The input graph network features are divided into training and validation sets to evaluate the link prediction methods. A 10-fold cross validation is carried out. Tables 9, 10 summarizes the 10-fold cross validation accuracies using the link prediction methods for the GDKG and DDKG networks, respectively. The average accuracies obtained for the gene-disease network link predictions are 93.07, 92.32, and 89.72% for the GNN, RF, and Gboost methods, respectively. The average accuracies obtained for the disease-drug network link predictions are 92.11, 92.63, and 91.62% for the GNN, RF, and Gboost methods, respectively. Overall, the GNN has the highest accuracy of 92.59%, followed by 92.48 and 90.67% for the RF and Gboost methods, respectively. The preferential attachment based link prediction gives an average accuracy of 83.92 and 67.06% for gene disease, and disease–drug link prediction, respectively. Here, we have combined the analysis of the six GeneLab GLDS datasets related to organ muscle atrophy in spaceflight. This is advantageous than analyzing them individually, as it reduces space and time complexity of processing. The three methods of RF, Gradient boosting, and GNN perform equally well, while the GNN shows a slightly higher accuracy.

**TABLE 3:** New muscle atrophy gene disease associations predicted by random walk network measure and GNN.

Gene name	Disease name	Link prediction
ATF3	Bone cancer and hypospadias	15
PTEN	Tumor suppressor	29
TNFRSF19	Ovarian cancer	11
BCL6	Lymphoma	8
EEF1B2	Seizures	17
UBASH3A	Type 1 diabetes mellitus	18
ZMIZ1	Neurodevelopmental disorder	32
PTPN22	Type 1 diabetes mellitus	22
AAMP	Tylosis With Esophageal Cancer	15
NOTCH1	leukemia	28
CDKN1B	Cell type cancer	8
FAM167A	Maturity-onset diabetes	11
DNMT3B	Immunodeficiency	15
ATF7IP	Testicular germ cell cancer	14
FADS1	Lipid metabolism disorder	18

*1 indicates existing association, 0 indicates predicted links.*

**TABLE 4:** Significant drugs used for treatment of diseases listed in Table 2.

Drugs				
Doxorubicin	Risperidone	Ivermectin	Rivastigmine	Streptokinase
Arcitumomab	Sertraline	Piperazine	Diethylcarbamazine	Cinnarizine
Nelarabine	Paroxetine	Suramin	Aminoglutethimide	Argatroban
Ifosfamide	Lamotrigine	Selegiline	Prednisolone	Nadroparin
Ixabepilone	Phenelzine	Albendazole	Certolizumab pegol	Lucinactant
Imatinib	Venlafaxine	Budesonide	Dichlorphenamide	Clindamycin
Etoposide	Isocarboxazid	Olsalazine	Fluocinolone acetonide	Telithromycin
Nimodipine	L-carnitine	Balsalazide	Loteprednol	Melatonin
Temozolomide	Thiamine	Verapamil	Antihemophilic factor	Amphetamine
Methotrexate	Galsulfase	Sulfasalazine	Anastrozole	Citalopram
Canagliflozin	Idursulfase	Warfarin	Acenocoumarol	Amisulpride
Insulin regular	Rosuvastatin	Azathioprine	Hydrocortisone	Fluvoxamine
Insulin lispro	Cysteamine	Infliximab	N-acetyl-d-glucosamine	Sulfapyridine
Insulin aspart	Icosapent	Golimumab	Indomethacin	Naproxen
Insulin glargine	Vitamin c	Adalimumab	Dihydrotachysterol	Tretinoin
Insulin, isophane	Riboflavin	L-arginine	Ergocalciferol	Ibuprofen
Insulin glulisine	Secretin	Hydralazine	Botulinum toxin type a	Mecasermin
Insulin detemir	Ketoconazole	Memantine	Desmopressin	Isopropamide
Pramlintide	Sulfisoxazole	Galantamine	Nandrolone decanoate	Inulin
L-carnitine	Tinidazole	Donepezil	Nandrolone phenpro.	Heparin
Secretin	Chloroquine	Tacrine	Glucagon recombinant	Mifepristone
Fluoxetine	Tetracycline	Vitamin e	Nandrolone phenpro.	Diazoxide
Insulin lispro	Icosapent	Daunorubicin	Sulfinpyrazone	Colchicine
Allopurinol	Sulindac	Etoricoxib	Flumethasone pivalate	Dronedarone
Cinacalcet	Dantrolene	Celecoxib	Antihemophilic factor	Tyloxapol
Maprotiline	Icosapent	Dornase alfa	Nandrolone decanoate	Urokinase
Insulin lispro	L-ornithine	Daunorubicin	Sulfinpyrazone	Colchicine
Probenecid	Allopurinol	Sulindac	Etoricoxib	Naproxen
Dronedarone	Secretin	Cinacalcet	Dantrolene	Celecoxib
Prednisone	Tyloxapol	Ivacaftor	Agalsidase beta	Dornase alfa
Enoxaparin	Alendronate	Probenecid	Calcium acetate	Ivacaftor
Insulin lispro	L-ornithine	Daunorubicin	Sulfinpyrazone	Colchicine
Probenecid	Allopurinol	Sulindac	Etoricoxib	Naproxen
Dronedarone	Secretin	Cinacalcet	Dantrolene	Celecoxib
Prednisone	Tyloxapol	Ivacaftor	Tranylcypromine	Dornase alfa
Enoxaparin	Alendronate	Probenecid	Aminoglutethimide	Ivacaftor

**TABLE 5:** Possible drugs for repurposing for muscle atrophy treatment predicted by random walk network feature and GNN.

Disease name	Repurposed drugs	Probabilities
Integumentory system cancer	Arcitumomab	90.52
Hypertension	L-arginine	89.44
Type 2 diabetes mellitus	Insulin	88.980385
Cardiovascular system disease	Selegiline	87.93291
Ulcerative colitis	Infliximab	87.20253
Autonomic nervous system neoplasm	Loteprednol	87.038925
Hematologic cancer	L-ornithine	86.95308
Ulcerative colitis	Olsalazine	86.79947
Vitiligo	Loteprednol	86.54014
Crohn’s disease	Infliximab	86.485664
Gastrointestinal system cancer	Golimumab	86.456894
Colitis	Olsalazine	86.39726
Colitis	Azathioprine	86.141464
Intestinal benign neoplasm	Olsalazine	86.11937
Inflammatory bowel disease	Hydrocortisone	86.08773
Crohn’s disease	Olsalazine	85.965416
Intestinal cancer	Olsalazine	85.94351
Crohn’s disease	Balsalazide	85.63084
Colorectal cancer	Hydrocortisone	85.55948
Systemic scleroderma	L-arginine	85.43495
Crohn’s disease	Certolizumab pegol	85.31858
Gastrointestinal system disease	Balsalazide	85.06852
Dermatitis	L-ornithine	84.991
Gastrointestinal system disease	Certolizumab pegol	84.908
Gastrointestinal system benign neoplasm	Balsalazide	84.827
Crohn’s disease	Budesonide	84.744
Muscular disease	L-ornithine	84.532
Demyelinating disease	Tinidazole	83.901
Demyelinating disease	Ivermectin	83.811
Leprosy	Tetracycline	83.778
Peripheral artery disease	Riboflavin	83.776
Nasal disorder	Tetracycline	83.659
Muscular disease	Adalimumab	83.642
Reproductive organ cancer	Tetracycline	83.485
Hematologic cancer	Nimodipine	83.203
Autoimmune disease of the nervous system	Ivermectin	83.107
Testicular cancer	Tinidazole	82.969
Testicular cancer	Sulfisoxazole	82.877
Neurodegenerative disease	Tinidazole	82.860
Neurodegenerative disease	Tetracycline	82.828

**TABLE 6:** Network measures for possible drug treatments for muscle atrophy in spaceflight.

Drug name	Degree distribution	Neighborhood connectivity	Subgraph centrality
Adalimumab	42	9.76	437,551.8
Arcitumomab	60	9.83	652,802.9
Azathioprine	14	10	6,881.267
Balsalazide	14	10	6,881.267
Budesonide	14	10	6,881.267
Certolizumab pegol	14	10	6,881.267
Golimumab	14	10	6,881.267
Hydrocortisone	14	10	6,881.267
Infliximab	14	10	6,881.267
Insulin	6	10	39.17687
Ivermectin	26	10	5,830.759
L-arginine	30	10	258,302.4
L-ornithine	5	10	79,395.46
Loteprednol	40	10	469,331.1
Nimodipine	48	9.79	402,801.8
Olsalazine	14	10	6,881.267
Riboflavin	31	10.45	79,395.46
Selegiline	45	10	523,266.8
Sulfisoxazole	26	10	138,229.6
Tetracycline	26	10	138,229.6
Tinidazole	26	10	138,229.6

**TABLE 7:** Network measures for the GDKG and DDKG networks.

Network measure	GDKG	DDKG
Spectral gap	9.015	1.011
Density	0.027	0.048
Average number of neighbors	7.993	11.178

**TABLE 8:** Results using Graph Neural Network (GNN), Gradient Boosting (GB), and Random Forest (RF) for link prediction in the Gene Disease Knowledge Graph (GDKG) and Disease Drug Knowledge Graph (DDKG).

	GDKG			DDKG		
	GNN	RF	GB	GNN	RF	GB
TP	448	476	466	235	254	249
TN	196	159	145	272	262	262
FP	34	71	85	9	21	21
FN	56	28	38	47	40	45

*Table shows number of True Positive (TP), True Negative (TN), False Positive (FP), and False Negative (FN) rates.*

**TABLE 9:** Ten-fold cross validation accuracies for link prediction using RF, Gboost, and GNN in GDKG.

Methods	1	2	3	4	5	6	7	8	9	10	AUROC
RF	92.17	93.65	95.91	92.66	91.55	96.59	91.13	92.57	92.49	90.55	92.32
Gboost	87.86	90.56	89.32	88.78	88.25	93.19	86.07	90.55	88.34	88.75	89.72
GNN	95.79	95.20	96.38	89.58	94.50	95.86	96.44	95.30	94.60	91.69	93.07

**TABLE 10:** Ten-fold cross validation accuracies for link prediction using RF, Gboost, and GNN in DDKG.

Methods	1	2	3	4	5	6	7	8	9	10	AUROC
RF	91.20	86.69	88.77	93.09	88.76	93.55	89.48	92.74	86.13	88.30	92.63
Gboost	91.49	89.54	91.46	91.78	89.70	94.23	90.09	93.58	88.74	89.68	91.62
GNN	88.15	87.80	88.67	92.00	88.81	90.91	91.01	89.11	87.39	86.95	92.11

## Discussion

The shared key genes from the Markov Blanket GRN of all the six GeneLab datasets with maximal differential regulation are given in Table 1. Figure 3 shows the GDKG constructed using the top regulated genes from the six GeneLab datasets and the SPOKE database. The red nodes represent the genes, and the blue nodes represent diseases. Table 2 lists the disease nodes present in Figure 3. Table 3 lists 15 new gene disease associations predicted by the GNN link prediction method. There are several differentially regulated genes resulting in reduced proliferation of thymic cells, thereby reducing the size of the thymus (Horie et al., 2019a). Of these the ATF3 is a key gene player identified in Table 1. This gene encodes a member of the mammalian activation transcription factor and is induced by a variety of signals, including many of those encountered by cancer cells. It is involved in the complex process of cellular stress response. This gene has 15 additional links predicted by the GNN. PTEN is an important gene that suppresses cell growth into tumors, which has been identified as a key gene in the GDKG. This gene is found to regulate muscle protein degradation in diabetes (Hu et al., 2007). In the GDKG network this gene has 33 existing links, and 29 new links to existing diseases are predicted. Tumor Necrosis Factor (TNF) is one of the most important muscle-wasting cytokine, elevated levels of which cause significant muscular abnormalities (Bhatnagar et al., 2010). The protein encoded by TNFRSF19 is a member of the TNF-receptor family. When overexpressed it activates the JNK signaling pathway. The diseases associated with this gene are ovarian cancer and ectodermal dysplasia (Dostert et al., 2019). This gene originally had nine links in the GDKG, and eleven new links were added by the GNN link prediction method implying the importance of this gene in muscle atrophy prognosis in spaceflight. The BCL6 gene is a regulator of T-cell-dependent inflammation and autoimmune responses. BCL6 is likely to regulate B and T-cells via cell-specific biochemical mechanisms. Dysregulation of BCL6 could contribute to BCL6+ T-cell lymphomas and regulated in urinary bladder urothelial carcinoma (Wu et al., 2020). This gene has eight existing links and eight links have been added by the link prediction method, showing the importance of this gene in spaceflight induced muscle atrophy. The EEF1B2 gene encodes a translation elongation factor specifically expressed in neurons and muscles (Doig et al., 2013). The protein is a guanine nucleotide exchange factor involved in the transfer of aminoacylated tRNAs to the ribosome. Diseases associated with EEF1B2 are seizures, alacrima, achalasia, and intellectual instability syndrome. This gene has seven existing links in the GDKG, and 17 new predicted links. Apart from these five key genes there are 10 more mentioned in Table 3. The network measures for these 15 genes are listed in Table 1. Compared to the other genes in Table 1, these 15 genes with higher number of predicted links also have higher values of degree distribution, neighborhood connectivity, and subgraph centrality network measures, as listed in Table 1. These genes also have higher link prediction probabilities greater than 90%. The diseases associated with these genes are cancer, diabetes, and neurological disorders most of which have muscle atrophy as a side effect. Prolonged exposure to spaceflight may cause risk of contracting these diseases. Hence, preventive medicine and therapeutics are key in warding off these conditions.

### Implications for Spaceflight

Several spaceflight experiments have shown that changes in the physical environment modulate cellular responses thus accelerating the risk of age-related diseases such as bone loss, muscle atrophy, and impaired immune responses (Versari et al., 2013; Cadena et al., 2019). Investigations on muscle atrophy in organs and tissues including cutaneous muscles in rodent and human models are being conducted in spaceflight for over a decade (Däpp et al., 2004; Neutelings et al., 2015; Goropashnaya et al., 2020). There are about 20 datasets available in GeneLab on muscle atrophy investigation on animal models in spaceflight (NASA Gene Lab data repository, n.d.). Formeterol is the only drug tested so far in spaceflight to mitigate muscle atrophy in mice (Ballerini et al., 2020). While experimental drug repurposing and clinical testing are prolonged and expensive, our proposed network science and artificial intelligence framework is computationally inexpensive and can be used for the rapid selection of candidate drugs to treat muscle atrophy in spaceflight. As muscle atrophy is a condition caused by many terrestrial diseases, the medications prescribed for these diseases can be useful candidates for repurposing for muscle atrophy. Hence, we constructed the GDKG for muscle atrophy to determine the diseases that have muscle atrophy as a primary side effect, and performed link prediction to identify the drugs that treat these diseases and can be repurposed for treating muscle atrophy. Figure 4 shows the DDKG constructed from the top ranked gene diseases associations from the GDKG, and the drugs used in treating these diseases. The blue nodes represent diseases, and the purple nodes represent the drugs. Table 4 lists the drugs from the network in Figure 4. The three link prediction algorithms are applied to the DDKG for identifying possible drugs for muscle atrophy treatment. Table 5 lists the drugs with probabilities higher than 80%. These drugs are used for treating the conditions that have muscle atrophy as a severe side effect such as cancer, diabetes, and nervous system disorders. For example, antidiabetic agents such as metformin, incretins, vitamin D, formoterol are medications that can reduce muscle wastage while treating diabetes (Campins et al., 2017). Indeed the GeneLab datasets GLDS-244 and GLDS-245 were collected to evaluate the efficacy of the drug formoterol to treat muscle atrophy in spaceflight flown mice (Ballerini et al., 2020). Muscle loss is also present in Chronic Obstructive Pulmonary Disease (COPD). The medication bimagrumab that treats COPD also resulted in increase in thigh muscle volume. By constructing the DDKG and applying link prediction, we have identified drugs belonging to the Monoclonal AntiBodies (MABs) family that are used for treating cancer as promising candidates for muscle atrophy in spaceflight. These include adalimumab, arcitumomab, certolizumab, golimumab, and infliximab. Table 5 lists the probabilities for these drugs as well as others that treat cancer and other diseases. Hence, one drug is repeated several times in Table 5. In total, there are 21 drugs that have higher probabilities for predicted links. The network measures for these drug nodes in the DDKG network is listed in Table 6. As can be seen, all of these drugs have similar values for degree distribution, and have a neighborhood connectivity between 9 and 10. The drugs with highest measures for degree distribution, neighborhood connectivity, and subgraph centrality are Nimodipine, Arcitumomab, Selegiline, Tetracydine, and Loteprednol. Arcitumomab, L-Arginine, L-Ornithine, and Nimodipine which are used for treating cancer and muscle disuse. Selgiline is used for treating cardiovascular diseases. Most of the 21 drugs that can be repurposed for muscle atrophy treat some type of cancer. The repurposing of a drug to treat muscle atrophy is limited by the drug database as the condition itself is secondary to diseases that have no cures. The selection of drugs to treat muscle atrophy in spaceflight could be based on those that can provide clear cures and can be effectively repurposed.

### Network Analysis

Table 7 lists the network measures of girth, density and spectral gap for the GDKG and DDKG networks. As can be seen from these measures the GDKG network has higher spectral gap of 9.015. The larger the spectral gap (the smaller | λ_2_|), the higher the network flow with sparseness, expansion, diffusion, and random walk. Hence, these networks have a higher measure of random walks, implying that the nodes that lie closer to each other in the network perform similar functions. The advantage of using networks and AI methods for drug repurposing is that the graphs themselves are scalable and can include more genes, disease, and drug nodes and the deep learning architecture can be built to handle corresponding large scale prediction problems. The network sciences approach and the AI based tool can be used to predict key targets and potential diseases arising from spaceflight missions and will facilitate countermeasure development.

### Key Genes Description

Table 1 lists the highly activated genes from the spaceflight mice muscle atrophy datasets. These genes are involved in protein amino acid binding, glycoprotein binding, cell growth and/or maintenance, and cell adhesion receptor inhibitor activity. These genes are part of cellular metabolic pathways by which individual cells transform chemical substances and pathways involving organic or inorganic compounds that contain nitrogen. They are also involved in chemical reactions and pathways involving an organic substance, any molecular entity containing carbon, and in chemical reactions and pathways involving those compounds which are formed as a part of the normal anabolic and catabolic processes. Some of these genes are involved in organ system process carried out by any of the organs or tissues of the neurological system. 15 key genes with the highest number of newly predicted links is given in Table 3 and their associated diseases from diseases from GeneCards (2021) is also given here. As can be seen half of these genes are associated with some type of cancer, followed by diabetes.

## Conclusion

We have presented a novel method for generating GDKGs for a particular disease from gene expression datasets using network analysis and the SPOKE database. In this research, we have worked with transcriptional gene expression datasets for muscle atrophy in mice flown in spaceflight microgravity. Link prediction applied to this network reveals interesting relationships of key genes with different types of cancer. The link prediction method is also used on the Disease Drug Knowledge Graph resulting in the identification of novel drugs that are possible candidates for treating muscle atrophy accelerated due to spaceflight travel. We have combined six GeneLab datasets in an innovative way with disease and drug databases and applied network analysis and artificial intelligence methods for drug repurposing.

## Data Availability Statement

Publicly available datasets were analyzed in this study. This data can be found here: genelab.nasa.gov.

## Author Contributions

VM: conceptualization, supervision, project administration, and funding acquisition. VM, JO-S, and VD-M: methodology and formal analysis. JO-S and VD-M: software, validation, data curation, and visualization. VM and JO-S: investigation and writing—original draft preparation, review, and editing. All authors have read and agreed to the published version of the manuscript.

## Conflict of Interest

The authors declare that the research was conducted in the absence of any commercial or financial relationships that could be construed as a potential conflict of interest.

## Publisher’s Note

All claims expressed in this article are solely those of the authors and do not necessarily represent those of their affiliated organizations, or those of the publisher, the editors and the reviewers. Any product that may be evaluated in this article, or claim that may be made by its manufacturer, is not guaranteed or endorsed by the publisher.

## Data Availability

This research was funded by NASA EPSCoR (Grant Number 80NSSC20M0132). Opinions, findings, conclusions, or recommendations expressed in this material are those of the authors and do not necessarily reflect the views of NASA.

## Footnotes

1
https://www.mayoclinic.org/diseases-conditions/muscular-dystrophy/symptoms-causes/syc-20375388

2
https://go.drugbank.com/

3
https://github.com/jjorozco777/An-integrative-Network-Science-and-Artificial-Intelligence-Drug-Reporposing-Approach
